# Rupture of Hydatid Cyst in the Gallbladder Leading to Acute Cholangitis

**DOI:** 10.1155/2021/9858658

**Published:** 2021-07-07

**Authors:** Hicham Elmajdoubi, Zakaria Elbarkaoui, Farid Sebbah, Mohamed Raiss, Abdelmalek Hrora

**Affiliations:** Surgery Unit C, Ibn Sina University Hospital, Rabat, Morocco

## Abstract

Hydatid disease is a health problem in endemic areas such as the Mediterranean region caused by *Echinococcus granulosus* which can develop anywhere in the human body, but it is most frequently located at the liver. Liver hydatid cyst may rupture into the biliary tract, thorax, peritoneum, viscera, digestive tract, or skin, but its rupture in the gallbladder remains rare. We report a rare case of rupture of liver hydatid cyst in the gallbladder leading to acute cholangitis. The diagnosis was suspected on radiological imaging, and the patient was taken to open surgery which confirmed the imaging findings. The gallbladder and adjacent cyst were excised, and a transcystic drain was placed. Postoperative recovery was uneventful.

## 1. Introduction

Hydatid disease is a nonimmunizing parasitic zoonosis caused by *Echinococcus granulosus*. The definitive host is usually a dog but may be some other carnivore. Humans are infected incidentally by food and water contaminated by tapeworm eggs. The liver is the most commonly involved organ by *Echinococcus granulosus* in the form of hydatid cyst which grows slowly.

Liver hydatid cyst can rupture in any part of the biliary system, but the communication with the hepatic bile ducts is most common. Rupture between a hepatic hydatid cyst and the gallbladder is rare [1].

We present a case of rupture of liver hydatid cyst in gallbladder with dilatation of the intrahepatic biliary tree.

## 2. Case Report

A 60-year-old female with no significant medical history presented with right upper quadrant pain, icterus with clay-colored stools, and itching for one month. The evolution was marked by fever and chills for 3 days. On admission, the patient's blood pressure was 120/80 mmHg, pulse was 84 beats/min, and temperature was 38.5°C. Physical examination revealed tenderness in the epigastric region and right upper quadrant of the abdomen. There was no hepatomegaly or splenomegaly, and the rest of the clinical examination was without particularities.

The laboratory examinations on admission showed white blood cell count (WBC) of 13000/*μ*L, C-reactive protein (CRP) 15 mg/L, serum glutamic-oxaloacetic transaminase (SGOT) 37 *μ*/L, serum glutamic pyruvic transaminase (SGPT) 37 *μ*/L, and total bilirubin 250 mg/L with direct bilirubin 150 mg/L. The patient was diagnosed with cholangitis due to the presence of pain, fever, and jaundice. Radiological examinations were applied to identify the etiology. Abdominal ultrasound revealed a cystic lesion at segment IV of the liver. This formation measures 67/44 mm, and it seems to communicate with the gallbladder which contains multiple stones with compression leading to dilatation of the intrahepatic bile ducts.

**Figure 1:**
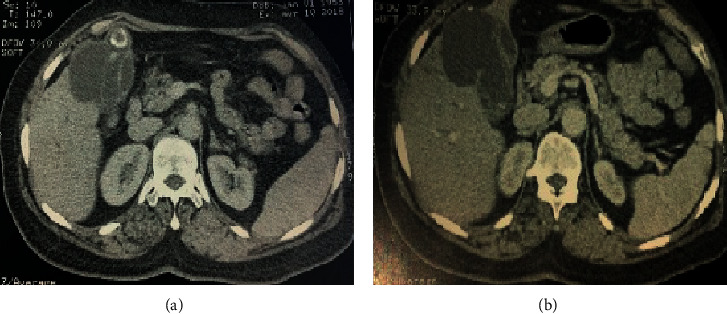
(a, b) Computed tomography (CT) scan showing a fistula between hydatid cyst and gallbladder.

Abdominal CT scan was performed for a better characterization and showed hydatid cyst at segment IV of the liver fistulized in the gallbladder which is multilithiasic with dilatation of the intrahepatic biliary tree (Figure 1).

**Figure 2:**
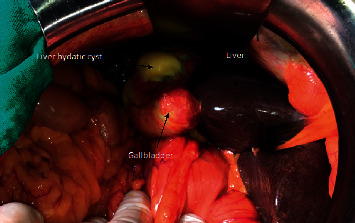
Operative image showing a hydatid cyst of segment IV adherent to the gallbladder.

During treatment, the patient was given 10 mg/kg a day of albendazole for hydatid cyst treatment; 1 g (2 × 1) ceftriaxone, and 500 mg (3 × 1) metronidazole were initiated for cholangitis treatment. The patient underwent right subcostal laparotomy which revealed a hydatid cyst of segment IV adherent to the gallbladder with the cholestatic liver. The exploration did not show any other cystic lesions (Figure 2).

**Figure 3:**
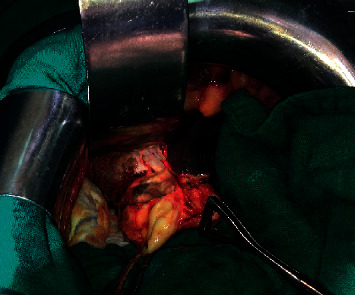
Operative image showing a fistulous communication between a liver hydatid cyst and the gallbladder.

**Figure 4:**
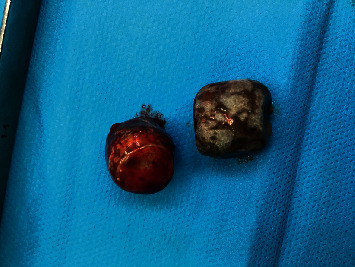
Gallstone removal from the gallbladder.

After using a scolicidal agent in the operating field, resection of the protruding dome along with cholecystectomy was performed (Figure 3). When the gallbladder was opened, macrolithiasis and daughter cysts were found (Figure 4) and the exploration of the residual cavity did not show any biliary fistula. A cholangiography by a transcystic drain showed dilatation of the intrahepatic bile ducts without dilatation of the main bile duct with passage of the product to the duodenum. Transcystic drain and epiploplasty with Redon drainage were performed.

The patient's postoperative course was uneventful, and she was discharged on the 6th postoperative day. Histopathologic examination showed a hydatid cyst with fistula to the gallbladder as we suspected.

The patient received albendazole 400 mg twice daily for three cycles. After six months of clinical and ultrasonographic follow-up, no recurrence occurred.

## 3. Discussion

Hydatid disease is caused by *Echinococcus granulosus* or *Echinococcus multilocularis* and affects the liver in 70% of cases [2, 3]. It is still endemic in Morocco and represents a health problem in developing countries.

The liver (70–80%) and lungs (15–25%) are the most common sites of hydatid cysts, but involvement of other organs is also possible. The exact incidence of extrahepatic cysts is not known [4]. The frank biliary communication has been reported in only 5–15% of cases [5]. It occurs in the right duct in 55–60% of cases, in the left duct in 25–30%, and rarely in the biliary confluence or gallbladder [6]. Our patient has a hydatid cyst of segment IV open in the gallbladder.

Clinical presentation consists generally pain localized in the right hypochondrium and rarely in the epigastrium [7]. Jaundice may occur by compression of the common bile duct by hydatid cyst or after migration of daughter cysts into the biliary tract. Our patient presented with right upper quadrant pain, fever, and jaundice.

Ultrasound and computed tomography are very helpful to diagnose hydatid cysts. They may detect hydatid disease in the form of purely cystic lesions or when floating membranes, daughter cysts, or vesicles are recognized [8]. Magnetic resonance imaging can be used in difficult cases, such as intrabiliary rupture, in which CT scan and sonography findings may be inconclusive [6].

The finding of bile duct dilatation in the vicinity of cystic lesions suggests bile duct compression or presence of a fistula opening into the bile ducts, which are events consistent with hepatic cystadenoma and hydatid cyst, respectively; cystic lesions of segment IV according to Couinaud's classification can lead to compression of the common hepatic duct and the right or left intrahepatic bile ducts [9]. In our case, there was a dilatation of the intrahepatic bile duct by compression, probably responsible for acute cholangitis.

Surgical intervention is the optimal treatment in hydatid cyst open in the gallbladder, and treatment options include the sterilization and drainage of hydatid cyst followed by radical (total cystopericystectomy) or conservative (partial pericystectomy) excision along with cholecystectomy. Following common biliary duct investigation and biliary lavage, T-tube drainage or transduodenal sphincteroplasty may be carried out, particularly if fistula is larger than 5 mm and if preoperative endoscopic retrograde cholangiopancreatography (ERCP) was not performed [10]. The preoperative ERCP obviates the necessity of choledochotomy and T-tube drainage by performing sphincterotomy or stent placement [11].

Albendazole or mebendazole should be used in the treatment of hydatid cysts either alone or as a preprocedure or postprocedure prophylaxis [12]. In our case, the patient received albendazole before and after surgery, resection of the protruding dome and cholecystectomy with transcystic drain were performed, and the postoperative course was uneventful.

## 4. Conclusion

Rupture of liver hydatid cyst in the gallbladder is extremely rare even in endemic areas. Treatment remains essentially surgical, and health education of the population in endemic areas remains the best mean of prevention.
